# The Registration of Midwives

**Published:** 1891-03-28

**Authors:** 


### The Registration of Midwives.

"Brisbane" writes: Having just read Dr. Haslam's "protest" regarding the registration of midwives, may I, as a midwife, make a few remarks upon it? The medical men and others who oppose the Bill persistently insist that "a new order" is being "created," and write as if at a given signal on the passing of the Bill, a flood of trained midwives (hitherto, I suppose, kept back) will be let loose on the land. Now no new order will be created by the passing of the Bill. Only an old order regulated. Midwives have been a recognised institution and necessity from the time of the birth of Esau and Jacob, and whether general practitioners like it or no, always will exist. It is estimated that at least 50 per cent. of the women in this country are delivered by women. Is this what Dr. Haslam calls a "limited demand" for midwives' services? Not a third of the number who attend as midwives have had any training. Alas! for their patients. I have until recently been working as district nurse and midwife in a rural district, under the shadow of the Black Mountains. The population, purely agricultural labourers, earning 12s. to 14s. a-week; the cottages very scattered. We were four miles from the nearest doctor (so much for the "vast machinery for giving the poor professional attendance in their own homes"; even the parish doctor was four miles off). In this district were three or four of these "handy-women." It was quite immaterial to them whether they attended a birth or laid out a corpse, and they would go from the latter to the former without changing their clothes or washing their hands. One of them gave out that a patient had been in labour four days. She then called in a doctor, putting the patient (a labourer's wife) to the expense of half a guinea, only to be told that labour had not commenced! I said to this woman, "Don't you examine your patients?" "Not I; I never touches 'em," was her reply. The same woman sent four miles for a doctor to remove a placenta that was lying in the vagina, and only required a lift to bring it. And in another case she let a primpara nearly lose her life from exhaustion, she not recognising that the head was arrested. All uterine action ceased, and it took all the doctor's skill, when he arrived, to save the patient. In this district only one doctor out of four was averse to a trained midwife. Then in country districts (I should think Dr. Haslam has had only town practice) there is the danger of loss of life from hæmorrhage, &c., before the doctor could possibly be got. I had one case of p.p. hæmorrhage in a stout woman with a weak heart, which must have proved fatal had I not known what to do, and had the necessary drugs at hand. And, in another, a woman was confined with a six and a-half months' child, which had been dead three weeks, breech presentation, and hydrocephalic head. Again, these women, in nine cases out of ten, would not recognise a malpresentation till they saw it, and when it would be most difficult for a doctor to rectify it. And yet, because it is wished to replace these women by others, who, even if of a "low grade" in obstetric practice, do know *something*, and to legislate so as to ensure that they are the best that can be provided, there is all this outcry. Doctors are eager enough to protect their own interests, but we midwives, who have paid heavily for our training, must not be accorded the same protection. Dr. Haslam speaks of midwives becoming possibly female abortionists. I have yet to learn that the number of midwives punished for this crime is in excess of the number of medical men (some of them qualified) who have been imprisoned for the same offence. I do not allow that the training in England, as far as it goes, has failed. One must be anything but an idiot to pass the examination of the Obstetrical Society, and fairly educated. And numbers do pass every examination. I quite grant that we are mentally capable of receiving and digesting higher training, such as is given abroad; but if this were mooted, oh, the groans there would be from Drs. Rentoul, Haslam, &c.! One word in favour of the doctors. I have a better opinion of their humanity than Dr. Haslam has, and I have never had to call upon one in an emergency in vain, even though in the middle of the night. Certainly, I take care not to call them up for a trifle, and always send word why I need their co-operation. Dr. Haslam's "protest" is a strange one. He wishes the untrained midwives entirely suppressed. They are quite three to one, at the lowest computation, to the trained ones. And yet he objects to the legislation which will, in time, fill their place with trained ones. That the poor law authorities will, when the Bill has passed, have to take the matter up, has, I think, been foreseen by the promoters of the Bill, both as regards training midwives at a cheaper rate in the infirmaries and providing them for their districts. The late Dr. Matthews Duncan spoke to me about it, some years ago. Finally, I would ask Dr. Haslam why such evil results should accrue from the passing of the Bill in England? We don't hear of them abroad, in the countries in which midwives have been for years under State control; and yet they are taught to be far more independent of medical aid than we are. It is to England's shame that she should be the only country in Europe that has no legislation in the matter, and that she should take so much rousing before she will see the cruel ills the poor suffer for want of it.

